# Early Recurrent Large Vessel Occlusion After Hemorrhagic Transformation in Newly Diagnosed Atrial Fibrillation: Challenges in Anticoagulation Timing and Repeat Mechanical Thrombectomy

**DOI:** 10.7759/cureus.114653

**Published:** 2026-08-17

**Authors:** Ahmed Elnour, Mohamed Ali, Khalid Ghalib, Naveed Sultan

**Affiliations:** 1 Internal Medicine, University Hospital Kerry, Tralee, IRL; 2 Internal Medicine, Cork University Hospital, Cork, IRL; 3 General Internal Medicine, University Hospital Kerry, Tralee, IRL

**Keywords:** atrial fibrillation, haemorrhagic transformation, intravenous thrombolysis, large vessel occlusion, mechanical thrombectomy

## Abstract

Atrial fibrillation (AF) is a major cause of cardioembolic stroke, and decisions regarding anticoagulation become particularly challenging when hemorrhagic transformation occurs after reperfusion therapy.

We report the case of a 76-year-old man who presented with an acute left middle cerebral artery (MCA) occlusion and underwent intravenous thrombolysis followed by successful mechanical thrombectomy. Subsequent imaging demonstrated hemorrhagic transformation of the infarcted area, and newly diagnosed AF was identified as the likely embolic source. Anticoagulation was deferred because of concerns regarding intracranial bleeding. One week later, the patient developed recurrent neurological deficits secondary to a new ipsilateral large vessel occlusion, requiring repeat mechanical thrombectomy. Following radiological stabilization of the hemorrhagic transformation, anticoagulation was initiated without further complications.

This case highlights the clinical challenge of balancing the risk of recurrent cardioembolic event against the risk of hemorrhagic progression when determining the timing of anticoagulation after AF-related ischemic stroke. It also demonstrates that repeat mechanical thrombectomy can be an effective treatment option for selected patients with recurrent large vessel occlusion.

## Introduction

Atrial fibrillation (AF) is responsible for approximately one-quarter of ischemic strokes and is associated with increased stroke severity, disability, and mortality [1]. Patients with AF-related stroke are at particularly high risk of recurrent embolic events during the early post-stroke period, making anticoagulation the cornerstone of secondary prevention [2,3]. However, determining the optimal timing of anticoagulation remains challenging when hemorrhagic transformation occurs following reperfusion therapy [4,5].

Hemorrhagic transformation is a well-recognized complication of intravenous thrombolysis and mechanical thrombectomy, particularly in patients with large infarcts and successful reperfusion [6,7]. While delayed anticoagulation may reduce the risk of intracranial hemorrhage expansion, it may simultaneously expose patients to recurrent cardioembolic events. Recent studies including the ELAN and TIMING trials [1,3,8,9] have provided guidance regarding anticoagulation timing after ischemic stroke, although patients with significant hemorrhagic transformation remain underrepresented [8].

We present a case of early recurrent large vessel occlusion in a newly diagnosed AF patient in whom anticoagulation was deferred because of hemorrhagic transformation following thrombolysis and mechanical thrombectomy, ultimately requiring repeat endovascular intervention.

## Case presentation

**Figure 1:**
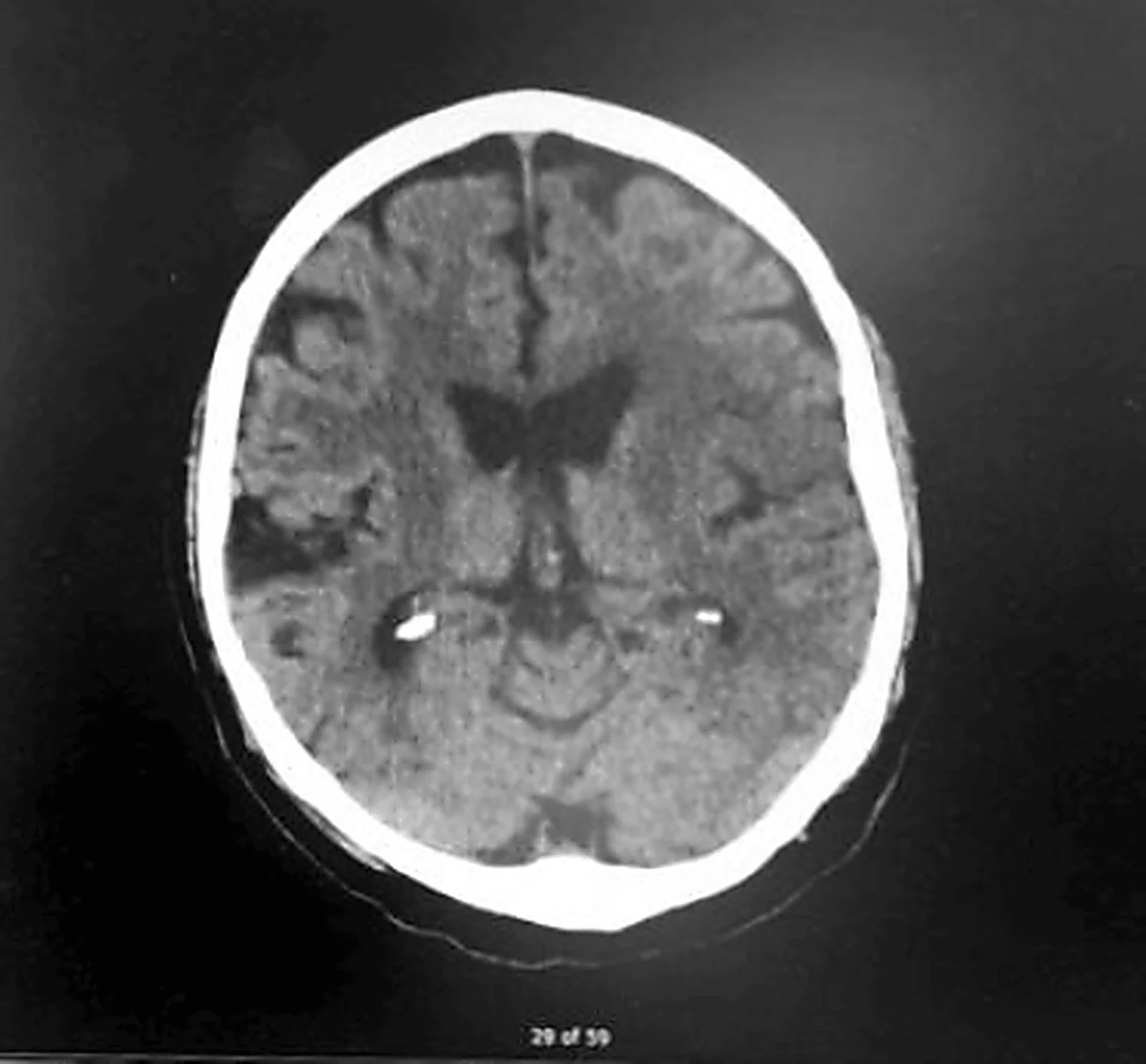
Non-contrast computed tomography (CT scan) No acute intracranial pathology identified; no evidence of intracranial hemorrhage, acute territorial infarction, space-occupying lesion, hydrocephalus, or midline shift.

A 76-year-old previously independent man with a history of hypertension and hyperlipidaemia presented to the emergency department 70 minutes after the acute onset of right-sided weakness, facial droop, and aphasia. On admission, his National Institutes of Health Stroke Scale (NIHSS) score was 17 (range, 0-42), consistent with a moderate-to-severe stroke. An urgent non-contrast computed tomography (CT) of the brain (Figure 1) demonstrated no evidence of acute intracranial haemorrhage.

**Figure 2:**
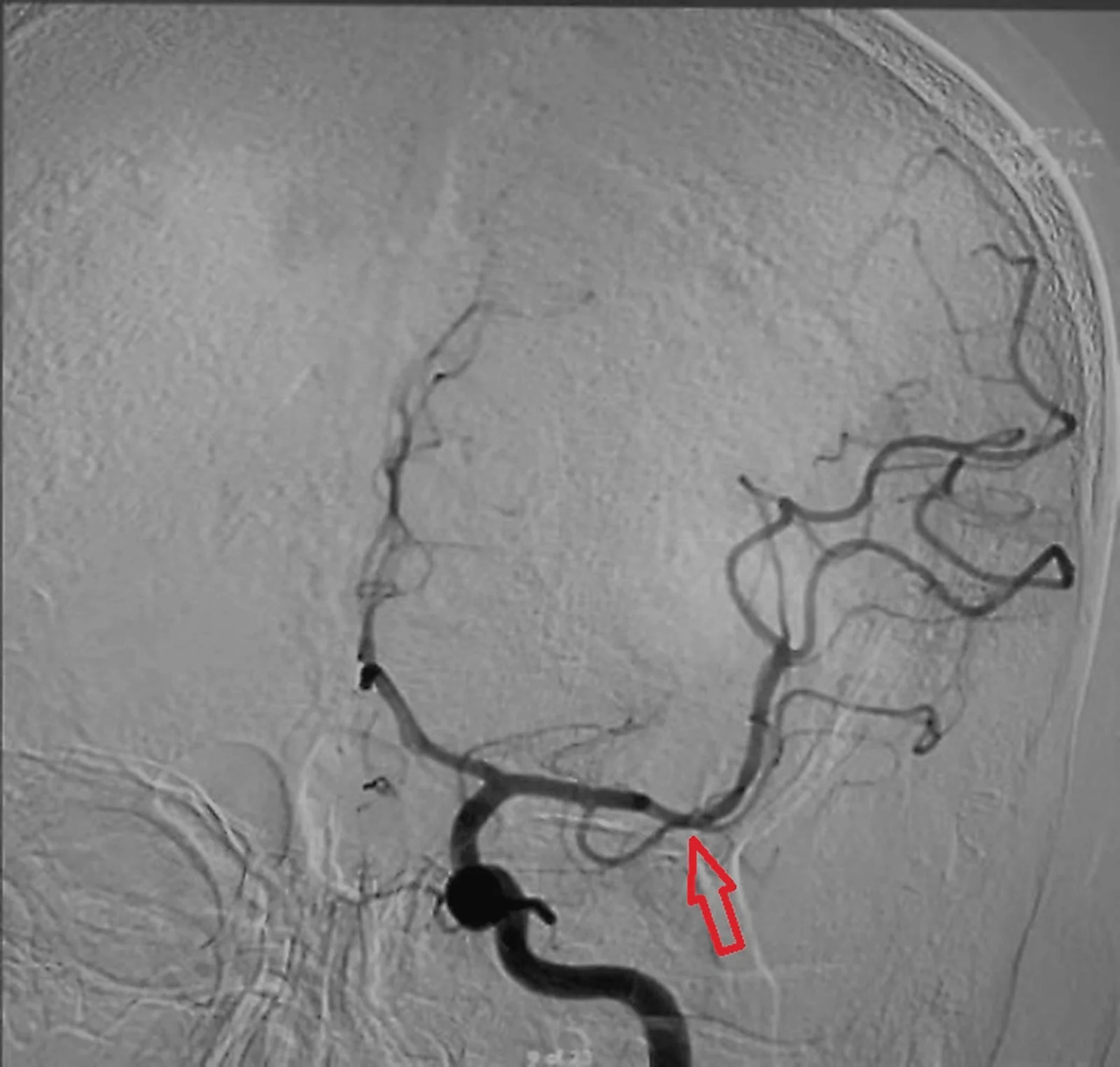
CT cerebral angiogram Initial cerebral angiography demonstrating left M2 middle cerebral artery occlusion (arrowed) prior to mechanical thrombectomy.

A subsequent CT cerebral angiography (Figure 2) revealed an occlusion of the left middle cerebral artery (MCA) at the M2 segment.

**Figure 3:**
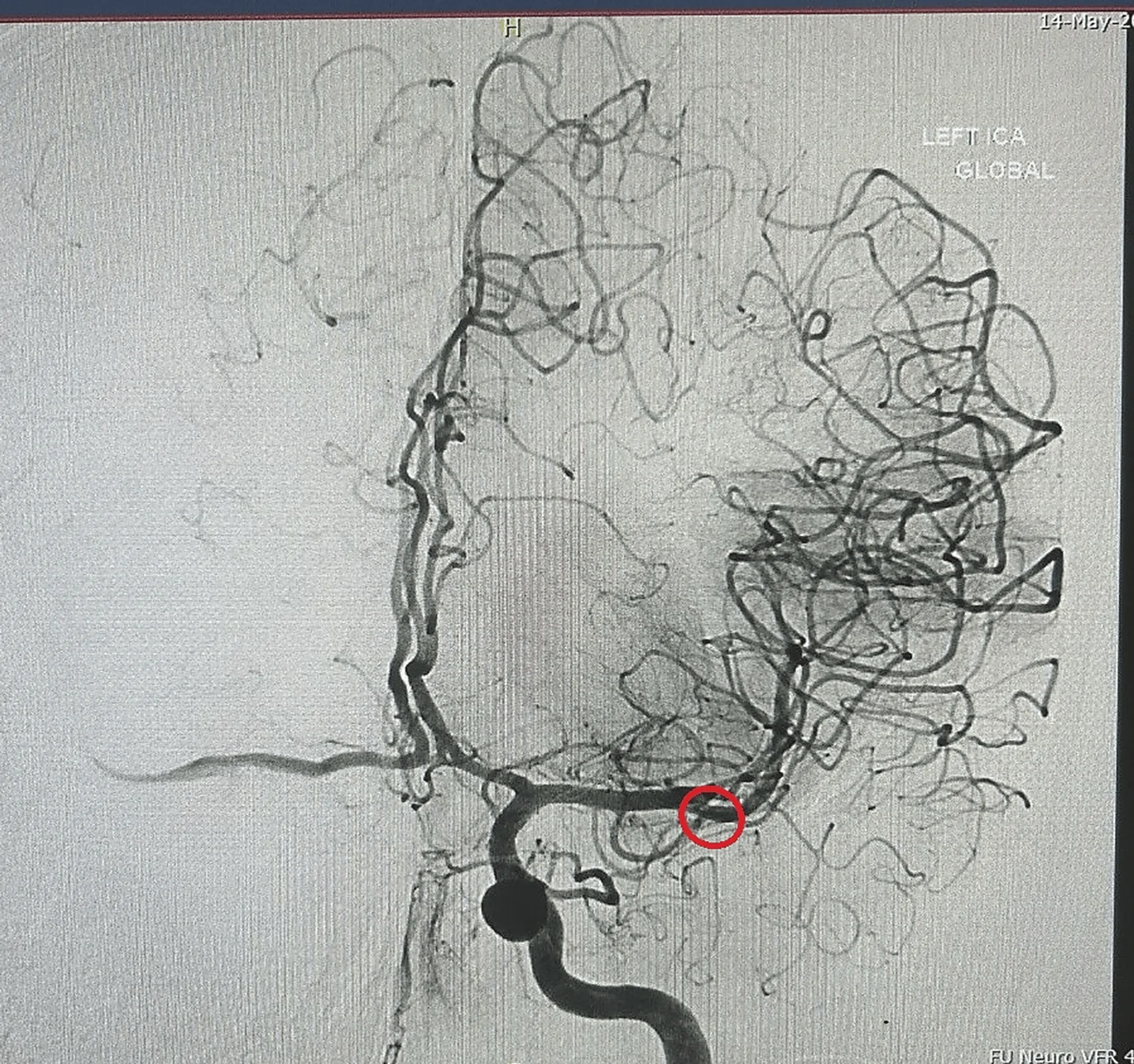
Post thrombolysis and thrombectomy CT cerebral angiography Cerebral angiography following thrombolysis and initial mechanical thrombectomy demonstrating successful reperfusion of the left middle cerebral artery (circled).

The patient received intravenous thrombolysis within the therapeutic window and subsequently underwent successful mechanical thrombectomy with significant reperfusion achieved (Figure 3).

**Figure 4:**
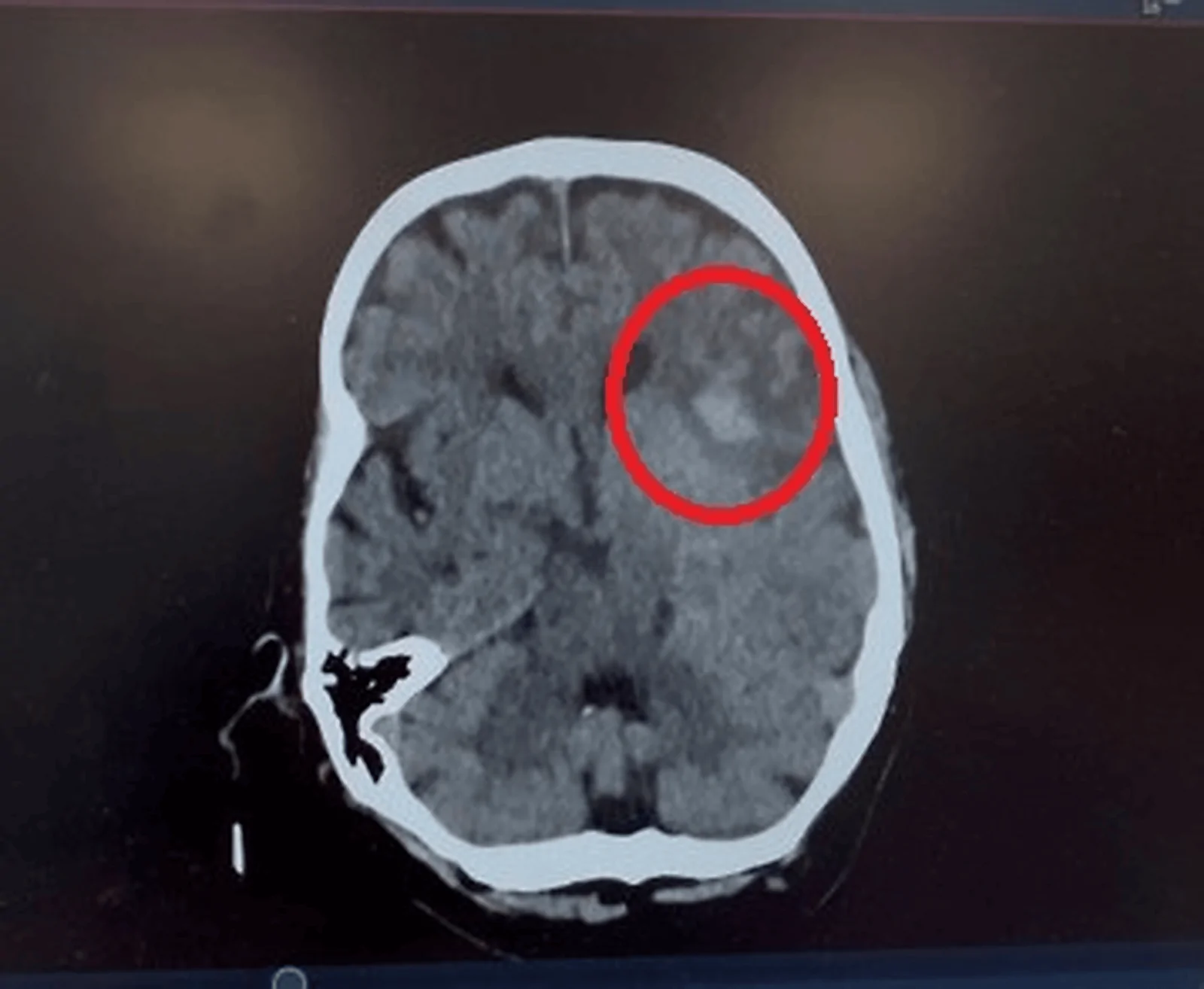
Post-thrombolysis and thrombectomy non-contrasted CT brain Post-reperfusion non-contrast CT brain demonstrating focal hemorrhagic transformation within the left middle cerebral artery infarct territory (red circle), without significant mass effect or midline shift.

Following the procedure, the patient was admitted to the stroke unit for monitoring and further management. A repeat routine CT brain scan performed after thrombectomy (Figure 4) demonstrated hemorrhagic transformation within the infarcted territory. The hemorrhage was judged to be secondary to reperfusion injury following thrombolysis and endovascular therapy.

**Figure 5:**
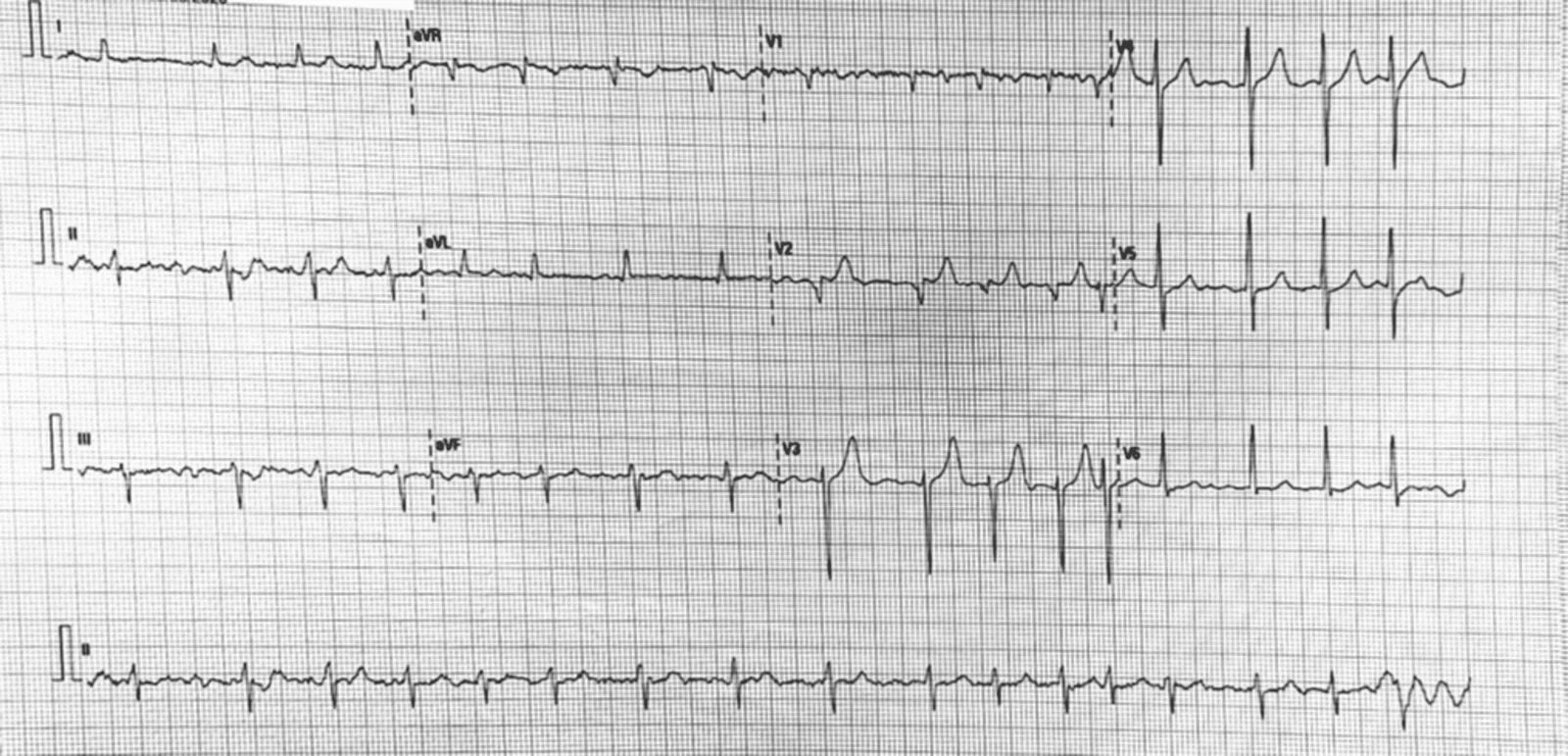
Electrocardiography (ECG) revealed atrial fibrillation ECG obtained during admission demonstrating newly diagnosed atrial fibrillation, considered the likely cardioembolic source of the ischemic stroke.

During admission, electrocardiography (ECG) revealed atrial fibrillation (Figure 5), considered the likely etiology of the stroke. Given the presence of hemorrhagic transformation, anticoagulation was deferred after multidisciplinary discussion involving stroke physicians and neuroradiologists due to concern regarding worsening intracranial bleeding.

**Figure 6:**
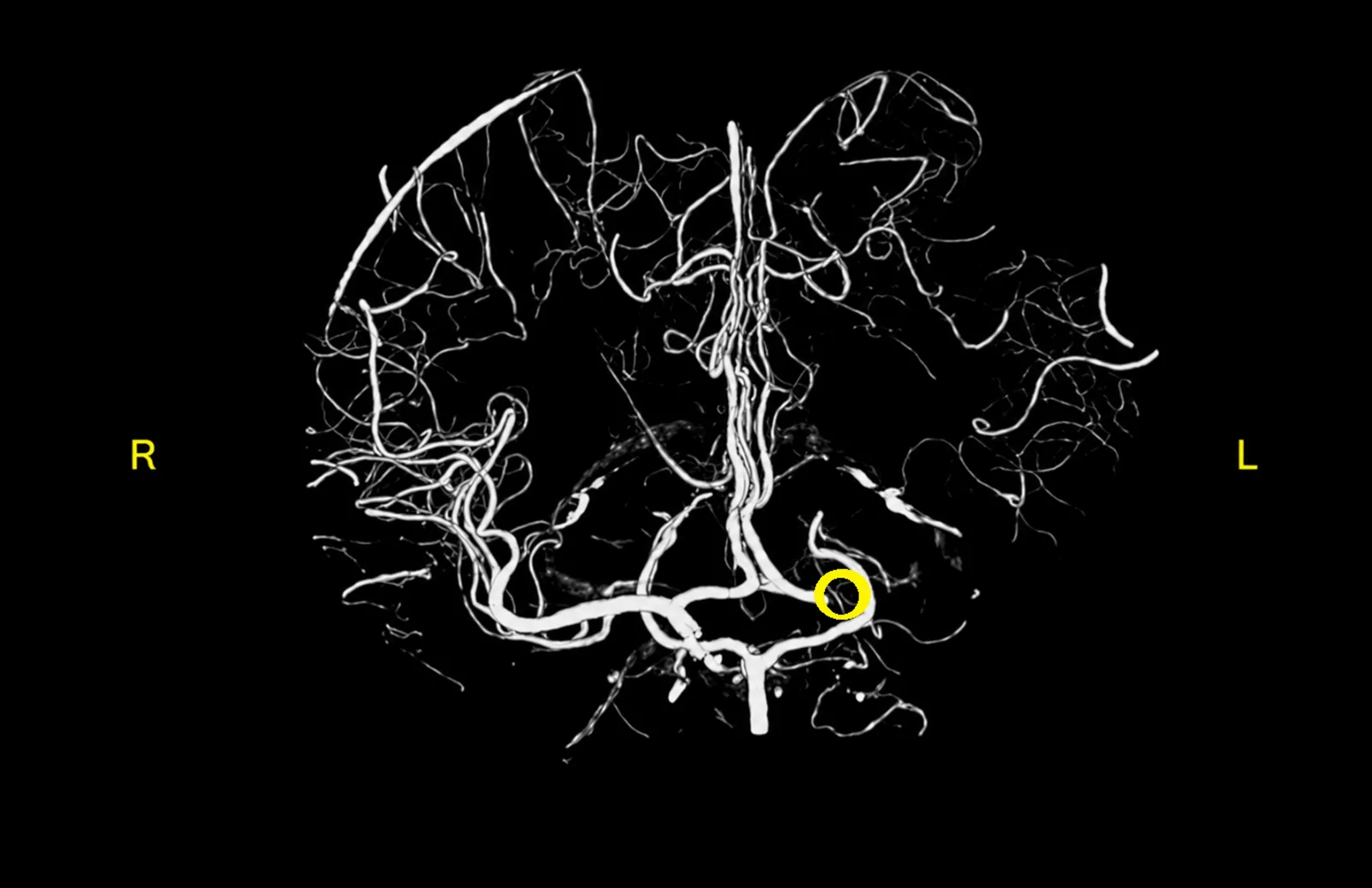
CT cerebral angiography Repeat vascular imaging following recurrent neurological deterioration approximately one week after the index stroke, demonstrating a new left M1 middle cerebral artery occlusion (yellow circle).

Following the initial mechanical thrombectomy, the patient showed marked neurological improvement, with his NIHSS score decreasing from 17 at presentation to 4. However, approximately one week after the index stroke, he experienced an abrupt deterioration characterized by recurrent right-sided weakness and worsening aphasia. His NIHSS score at that time was 14. An urgent repeat brain CT scan revealed a new large-vessel occlusion on the left side, involving the M1 segment of the left MCA (Figure 6).

**Figure 7:**
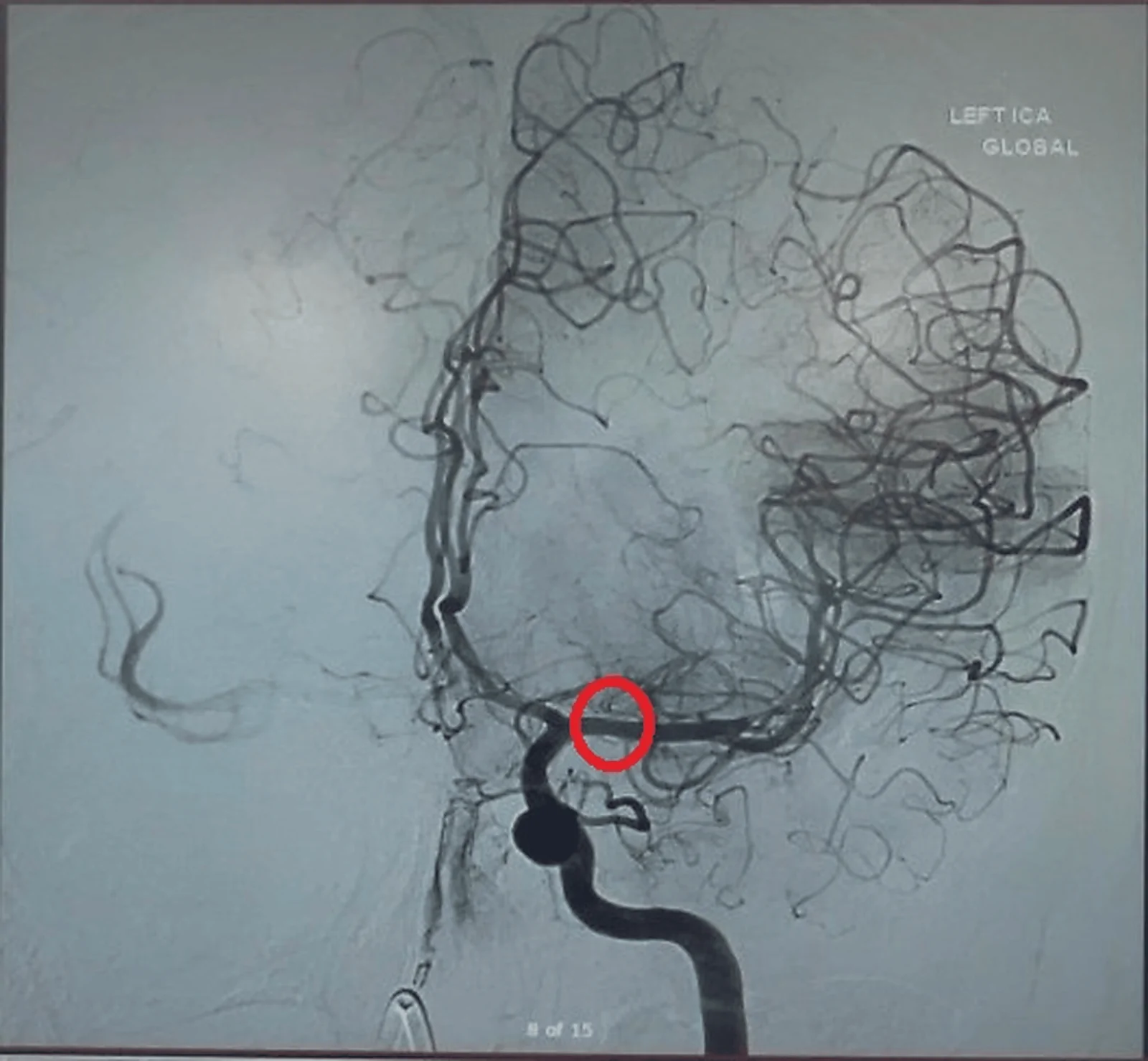
Post-thrombectomy CT angiogram Post-thrombectomy cerebral angiography demonstrating successful recanalization of the left M1 segment (circled) following repeat mechanical thrombectomy.

The patient underwent a repeat mechanical thrombectomy with successful recanalization (Figure 7). Post-procedure imaging showed no significant progression of intracranial hemorrhage. Following repeat mechanical thrombectomy and successful recanalization, the patient again demonstrated significant neurological improvement, with his NIHSS score decreasing from 14 before the procedure to 5 afterwards.

Following serial imaging demonstrating radiological stability of the hemorrhagic transformation, anticoagulation was initiated for secondary stroke prevention. The patient was discharged to a rehabilitation facility with ongoing physiotherapy, speech and Language therapy, and stroke follow-up.

## Discussion

This case highlights two clinically important issues: the challenge of determining the optimal timing of anticoagulation after atrial fibrillation-related ischemic stroke complicated by hemorrhagic transformation, and the role of repeat mechanical thrombectomy in the management of recurrent large vessel occlusion. The development of recurrent stroke within one week of the index event underscores the high early recurrence risk associated with untreated atrial fibrillation and the limitations of current evidence guiding anticoagulation decisions in this complex patient population.

AF-related ischemic stroke carries a particularly high risk of early recurrence, with observational data suggesting a recurrent ischemic stroke risk of approximately 0.5%-1.3% per day during the first two weeks in the absence of anticoagulation [1]. This risk is thought to reflect the persistence of the underlying cardioembolic source and is particularly relevant during the period in which anticoagulation must be delayed. Anticoagulation substantially reduces the risk of recurrent cardioembolism; however, early initiation may increase the risk of intracranial hemorrhage, particularly in patients with large infarcts or hemorrhagic transformation [7]. In our patient, this therapeutic dilemma was clinically evident: anticoagulation was initially deferred because of post-reperfusion hemorrhagic transformation, but recurrent large-vessel occlusion occurred approximately one week later.

AF is one of the most common causes of cardioembolic stroke and is associated with larger infarct volumes, greater neurological disability, and a higher risk of early recurrence compared with other stroke subtypes [2]

Hemorrhagic transformation is frequently observed after intravenous thrombolysis and mechanical thrombectomy, particularly in extensive infarcts with reperfusion injury [10]. The decision regarding anticoagulation timing in such cases remains individualized and often depends on infarct size, severity of hemorrhage, neurological status, and repeat imaging findings [3,5,8].

Several studies and trials have evaluated anticoagulation timing after ischemic stroke in AF. The ELAN trial suggested that early initiation of direct oral anticoagulants may be safe in selected patients [3,8]; however, patients with significant hemorrhagic transformation were generally underrepresented. Similarly, the TIMING trial supported earlier anticoagulation in carefully selected patients but emphasized individualized assessment [1,3,8].

Our patient presented a particularly challenging scenario because anticoagulation was withheld due to radiologically evident hemorrhagic transformation, yet he subsequently developed recurrent large vessel occlusion within one week. This highlights the high embolic potential associated with untreated AF and the limitations of current evidence in guiding anticoagulation decisions in complex cases.

Repeat mechanical thrombectomy has emerged as a feasible intervention in recurrent large vessel occlusion [10,11]. Although data remain limited, available evidence suggests that repeat thrombectomy may achieve favourable recanalization and clinical outcomes in selected patients [10,11].

This case emphasizes the importance of multidisciplinary decision-making involving stroke physicians, neuroradiologists, or cardiology teams when balancing hemorrhagic and ischemic risks.

## Conclusions

Patients with newly diagnosed atrial fibrillation following acute ischemic stroke remain at high risk of recurrent embolic events. Hemorrhagic transformation after thrombolysis and thrombectomy complicates decisions regarding anticoagulation timing and necessitates careful, individualized assessment.

This case illustrates the continuing risk of recurrent cardioembolic events during a period in which anticoagulation must be deferred because of hemorrhagic risk, without establishing a causal relationship between anticoagulation deferral and stroke recurrence. Multidisciplinary decision-making involving stroke physicians, neurologists, and neuroradiologists is essential for balancing these competing risks and guiding anticoagulation timing. Repeat mechanical thrombectomy may represent a viable therapeutic option in appropriately selected patients with recurrent large-vessel occlusion. Further research is required to establish optimal anticoagulation strategies in patients with hemorrhagic transformation after reperfusion therapy.
